# Use of AD Informer Set compounds to explore validity of novel targets in Alzheimer's disease pathology

**DOI:** 10.1002/trc2.12253

**Published:** 2022-04-12

**Authors:** Frances M. Potjewyd, Joel K. Annor‐Gyamfi, Jeffrey Aubé, Shaoyou Chu, Ivie L. Conlon, Kevin J. Frankowski, Shiva K. R. Guduru, Brian P. Hardy, Megan D. Hopkins, Chizuru Kinoshita, Dmitri B. Kireev, Emily R. Mason, Charles T. Moerk, Felix Nwogbo, Kenneth H. Pearce, Timothy I. Richardson, David A. Rogers, Disha M. Soni, Michael Stashko, Xiaodong Wang, Carrow Wells, Timothy M. Willson, Stephen V. Frye, Jessica E. Young, Alison D. Axtman

**Affiliations:** ^1^ UNC Eshelman School of Pharmacy Division of Chemical Biology and Medicinal Chemistry Structural Genomics Consortium Chapel Hill North Carolina USA; ^2^ UNC Eshelman School of Pharmacy Division of Chemical Biology and Medicinal Chemistry Center for Integrative Chemical Biology and Drug Discovery Chapel Hill North Carolina USA; ^3^ Department of Laboratory Medicine and Pathology University of Washington Seattle Washington USA; ^4^ Institute for Stem Cell and Regenerative Medicine University of Washington Seattle Washington USA; ^5^ Department of Medicine Division of Clinical Pharmacology Indiana University School of Medicine Indianapolis Indiana USA

**Keywords:** AD Informer Set, Alzheimer's disease, chemogenomic set, chemogenomics, target validation, Target Enablement to Accelerate Therapy Development for Alzheimer's Disease

## Abstract

**Introduction:**

A chemogenomic set of small molecules with annotated activities and implicated roles in Alzheimer's disease (AD) called the AD Informer Set was recently developed and made available to the AD research community: https://treatad.org/data‐tools/ad‐informer‐set/.

**Methods:**

Small subsets of AD Informer Set compounds were selected for AD‐relevant profiling. Nine compounds targeting proteins expressed by six AD‐implicated genes prioritized for study by Target Enablement to Accelerate Therapy Development for Alzheimer's Disease (TREAT‐AD) teams were selected for G‐protein coupled receptor (GPCR), amyloid beta (Aβ) and tau, and pharmacokinetic (PK) studies. Four non‐overlapping compounds were analyzed in microglial cytotoxicity and phagocytosis assays.

**Results:**

The nine compounds targeting CAPN2, EPHX2, MDK, MerTK/FLT3, or SYK proteins were profiled in 46 to 47 primary GPCR binding assays. Human induced pluripotent stem cell (iPSC)‐derived neurons were treated with the same nine compounds and secretion of Aβ peptides (Aβ40 and Aβ42) as well as levels of phosphophorylated tau (p‐tau, Thr231) and total tau (t‐tau) peptides measured at two concentrations and two timepoints. Finally, CD1 mice were dosed intravenously to determine preliminary PK and/or brain‐specific penetrance values for these compounds. As a final cell‐based study, a non‐overlapping subset of four compounds was selected based on single‐concentration screening for analysis of both cytotoxicity and phagocytosis in murine and human microglia cells.

**Discussion:**

We have demonstrated the utility of the AD Informer Set in the validation of novel AD hypotheses using biochemical, cellular (primary and immortalized), and in vivo studies. The selectivity for their primary targets versus essential GPCRs in the brain was established for our compounds. Statistical changes in tau, p‐tau, Aβ40, and/or Aβ42 and blood–brain barrier penetrance were observed, solidifying the utility of specific compounds for AD. Single‐concentration phagocytosis results were validated as predictive of dose–response findings. These studies established workflows, validated assays, and illuminated next steps for protein targets and compounds.

## 1 INTRODUCTION

The term “chemogenomics” has been associated with the use of small molecules to interrogate biology. This innovative technology has facilitated the identification of new therapeutic targets and accelerated target‐based drug discovery.^1^ A chemogenomic set is described as a collection of small molecules with annotated and narrow activity.^1^, ^2^ Use of such a compound library in a phenotypic screen allows correlation of an observed phenotype with pharmacologic perturbation of an annotated target of the hit compound. Based on the results, mechanistic hypotheses can be generated and follow‐up studies initiated to further validate a target. Target validation experiments will often rely upon target knockdown with gene silencing or editing technologies such as RNAi or CRISPR‐Cas9. When hits generated in a primary screen suggest that the target is amenable to functional pharmacological modulation, hits are advanced for chemical probe optimization in parallel.^1^ The integration of small molecule chemogenomics with target knockdown methods can result in new insights into biological targets and pathways in disease.

Since the earliest described chemogenomic sets, several focused libraries have been developed for protein target classes, such as kinases^2^, ^3^, ^4^ or epigenetic proteins.^5^, ^6^ More recently, these sets have been assembled with a therapeutic area focus, such as oncology.^7^, ^8^ A well‐designed chemogenomic set includes compounds that cover expansive pharmacological space. These can include Food and Drug Administration‐approved drugs as well as agents in clinical trials to provide opportunities for the repurposing of advanced candidates. Other favorable outcomes of using these sets have included new research findings, grants, and publications.^9^

The AD Informer Set was designed with many of these chemogenomics principles in mind and for a specific therapeutic area: hits from this set will point to potential targets driving phenotypic responses in AD‐relevant assays. This library of 171 small molecules targets 98 unique proteins that were nominated by the Accelerating Medicines Partnership Program for Alzheimer's Disease (AMP AD) consortium members and/or prioritized by the TREAT‐AD teams as novel targets for the treatment of AD. Multiple chemotypes targeting a single protein were included where possible as well as positive control compounds in advanced clinical trials or already approved for AD. Other important aspects of the AD Informer Set include comprehensive data annotation of compound‐ and gene‐specific attributes and open sharing of the set, allowing interested users to request it, use it without restrictions, and publish their findings.^10^

We selected central nervous system (CNS)‐relevant primary assays for selectivity profiling of AD Informer Set compounds. GPCRs are highly expressed, essential receptors in the brain involved in processes such as neuronal communication, neurogenesis, movement, and cognition.^11^, ^12^, ^13^ Given their abundance and importance, GPCRs could present potential off‐target liabilities or mediate confounding pharmacology for AD Informer Set compounds. While this provides preliminary characterization of the selectivity of specific compounds versus GPCRs, additional selectivity screening will be required to understand the comprehensive profiles of compounds within the set.

Human iPSC‐derived neural cells are increasingly being used as preclinical models in neurodegenerative research.^14^ Human iPSCs have the potential to fill a critical gap by providing live, functional human CNS cells with the complex genetic background found in AD patients. These cells create an important bridge between studies in animal models, assessment of human *post mortem* brain, and monitoring brain function in living patients.^15^

Pathophysiological hallmarks of AD include extracellular insoluble Aβ plaques and intracellular neurofibrillary tangles composed of hyperphosphorylated tau aggregates.^16^ Using the iPSC model system, Aβ secretion as well as tau and p‐tau expression can be measured as relevant AD readouts.

Blood–brain barrier (BBB) penetrance is required for a compound to be useful to AD patients. Measurement of the brain concentration of compounds with potential utility in AD where it has previously not been determined fills a critical gap. These data can support the advancement of an otherwise promising compound to in vivo target engagement and animal‐based AD models.

In the context of AD therapy, we are looking to identify compounds that can stimulate microglial phagocytosis with low or no associated cellular toxicity. The role of microglia in AD has been well documented and microglia act as mediators of neuroinflammation both early in AD and chronically as disease progresses.^16^, ^17^, ^18^, ^19^

## 2 METHODOLOGY

The intended uses of the AD Informer Set can be summarized as: (1) target validation in new and/or established AD models, (2) identification of positive controls and/or comparator compounds for benchmarking versus newly developed compounds, and (3) validation of newly developed and/or emerging AD‐relevant assays.^10^ With the goal of demonstrating that the AD Informer Set could prove useful in these specific contexts, we selected compounds from the set to execute several types of AD‐relevant assays and studies, including biochemical, cellular, and in vivo experiments. We added establishment of TREAT‐AD workflows and identification of key compounds as well as protein targets for follow‐up experiments as additional outcomes of these studies. We verified the utility of the set in AD‐relevant phenotypic assays designed to probe specific hypotheses and supplemented the annotation of specific compounds within the AD Informer Set with additional experimentally derived AD‐relevant data.

A set of nine AD Informer Set compounds was selected based on nomination of the gene by the AMP AD program coupled with heightened interest in the pathway by members of the TREAT‐AD consortium. These nine compounds target the proteins expressed by six genes: *CAPN2, EPHX2, MDK, MERTK/FLT3*, or *SYK*. Multiple exemplars built upon differing chemotypes were included for CAPN2 (2), EPHX2 (3), and SYK (2) proteins. The compounds selected vary in the amount of extant profiling and how advanced they are in development: dosed in animals and/or humans or an approved drug (see supporting information). In almost all cases, we evaluated responses to these compounds at multiple concentrations and/or timepoints.

**RESEARCH‐IN‐CONTEXT**

1. **Systematic review**: The authors used the recently assembled AD Informer Set in several experiments relevant to Alzheimer's disease (AD). Assays were selected to supplement available data for specific compounds within the set and validate therapeutic hypotheses related to their protein targets versus other important receptors in the brain.
2. **Interpretation**: This study provides proof‐of‐concept of the utility of the AD Informer Set as a chemogenomic set that can be used to validate novel targets in AD, qualify new AD‐relevant assays, and identify chemical starting points for optimization with AD therapy as the ultimate goal. We have implicated therapeutic directions for underexplored AD protein targets.
3. **Future directions**: This article provides a blueprint of how to use the AD Informer Set. Further studies can be aimed at: (a) screening the set in disparate assays; and (b) taking advantage of compound‐ or target‐specific drug discovery opportunities based on our results.

**HIGHLIGHTS**

- Utility of AD Informer Set was confirmed in proof‐of‐concept studies.
- Evaluation of AD Informer Set compounds in assays relevant to Alzheimer's disease (AD) was completed.
- AD phenotypes were associated with novel protein targets.
- Chemical starting points were identified for AD optimization.
- Compound‐specific data relevant to AD was generated and provided.

A second set of four AD Informer Set compounds was selected based on results from single‐concentration (10 μM) screening of the entire set.^10^ These compounds did not exhibit cytotoxicity but did exhibit a phenotypic response in preliminary microglial phagocytosis assays. Some cytotoxicity was observed with the prioritized nine compounds used in our other studies, so we opted to diversify our selection. The four compounds chosen target proteins expressed by four genes: *ACHE, ALK, CYP3A43*, or *ERBB3*. The compound targeting the ACHE pathway is an approved drug and was included in the AD Informer Set as a positive control compound. The other three compounds have also been dosed in humans, as one is in clinical trials while the other two are approved drugs (see supporting information).

## 3 RESULTS

A subset of nine AD Informer Set compounds prioritized by the Emory/Sage/Structural Genomics Consortium (SGC) TREAT‐AD team for in‐depth study were subjected to GPCR profiling, iPSC‐derived cellular assays, and mouse pharmacokinetic (PK) studies. In addition, non‐toxic compounds for which an interesting phenotype was observed in the phagocytosis assay were followed up in dose‐response. These studies were designed to align with our goal of demonstrating the utility of the AD Informer Set in three specific contexts. With respect to target validation in new and/or established AD models, we confirmed that inhibitors of the protein expressed by AMP AD–nominated target EPHX2 reduce tau phosphorylation in AD‐relevant neurons. EPHX2 inhibitor UNC10302681A/TPPU had previously been reported to prevent tau hyperphosphorylation in human nerve cells.^20^ To identify comparator compounds for benchmarking, we established off‐target GPCR profiles for two different compounds targeting SYK protein. Finally, aligned with validation of a newly developed AD‐relevant assay, AD Informer Set compounds were used to demonstrate the predictive value of single‐concentration data in our recently established microglial phagocytosis assay. Our studies provide real examples that help crystallize how the AD Informer Set can be used. They also establish workflows and benchmarks for AD chemical probe development within the Emory/Sage/SGC TREAT‐AD active target portfolio. Specific details, results, and significance of our work can be found in the sections that follow.

### 3.1 Data annotation

A spreadsheet with comprehensive annotation for compounds tested herein is included as supporting information. The second tab on this spreadsheet defines each column and summarizes the contents, including the meaning of abbreviations.

### 3.2 Microglial viability and phagocytosis studies

Four compounds (UNC10302865A/sapitinib, UNC10240506B/donepezil hydrochloride, UNC10100724A/ketoconazole, and UNC10244898A/lorlatinib) that were not cytotoxic at 10 μM and showed a phenotype (inhibition or activation)^10^ were followed up in dose‐response by the Chu lab. Assay details are included as supporting information. The single‐point data was found to be predictive of the dose‐response data in both cell lines. In addition, some toxicity was noted at higher concentrations in the expanded range up to 40 μM.

### 3.3 GPCR panel

A subset of nine selected compounds was submitted to the National Institute of Mental Health–sponsored Psychoactive Drug Screening Program (PDSP) at University of North Carolina (UNC). Details about the assays and data generated are included as supporting information files. Two compounds (UNC10302682A/P505‐15 and UNC2025C) possessed affinity for several receptors in the primary assays and were thus analyzed in those secondary assays. These secondary assays confirmed binding to only a portion of the receptors and that the corresponding affinities were modest. The remaining compounds did not exhibit notable affinity when profiled in secondary binding assays.

### 3.4 Human iPSC‐derived cellular assays

The same nine compounds were preliminarily analyzed by the Young lab in a panel of AD‐relevant assays. iPSC‐derived apolipoprotein E (*APOE*) ε3/ε4 neurons were differentiated and plated for assays as we have previously described^21^, ^22^ and outlined in the supporting information file. No evidence of cytotoxicity resulted from treatment of these neurons with this subset of the AD Informer Set. We did not observe a change in the Aβ42:40 ratio upon treatment with any compound. For four compounds (UNC10302679A/GSK2256294A, UNC10302681A/TPPU, UNC10302683A/AR9281, and UNC10302680A/iMDK), we detected fluctuations in individual Aβ peptide levels at 24 hours that had resolved by 48 hours of treatment. Several compounds reduced the p‐tau:t‐tau ratio in the neurons, including UNC10302681A, UNC10302683A, and UNC10302680A when dosed at 1 μM for 24 hours. When the total levels of each peptide were examined, these compounds were found to decrease both p‐tau and t‐tau, but the decrease in p‐tau was more significant. We observed a reduction in the levels of p‐tau and t‐tau at 48 hours with both doses of SYK enzyme inhibitors (UNC10302682A/P505‐15 and UNC10302678A/entospletinib) but no change in the p‐tau:t‐tau ratio.

### 3.5 PK data

The nine‐compound subset was sent for mouse PK studies at Pharmaron. Mouse IV PK data had previously been published for three compounds.^23^, ^24^, ^25^ The remaining six compounds were sent for snapshot PK (see Table 2). Plasma plus brain concentrations were measured for the three published compounds and, based on PK snapshot results, three additional compounds. Experimental details for snapshot PK and brain concentration measurements are included as supporting information.

## 4 DISCUSSION

**FIGURE 1:**
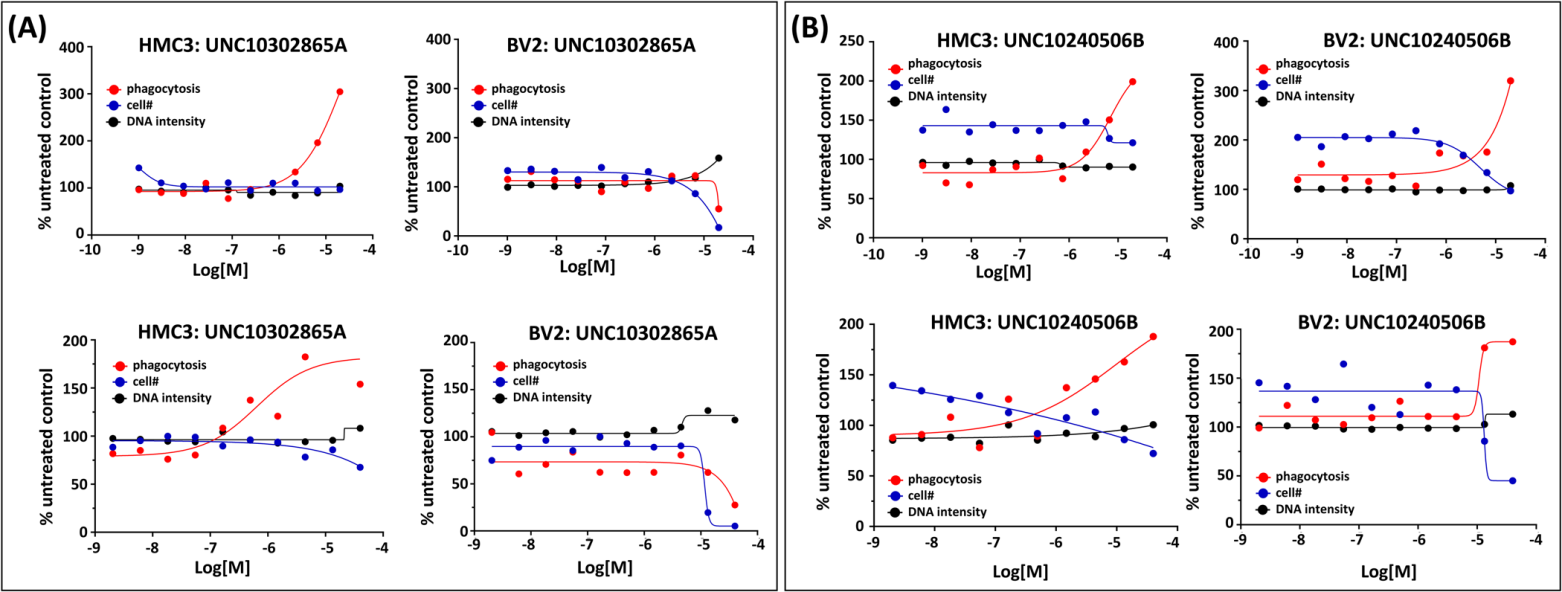
Phagocytosis assay 24 hour dose‐response follow‐up for (A) UNC10302865A and (B) UNC10240506B in HMC3 and BV2 cells

With respect to the microglia assays, UNC10302865A/sapitinib, UNC10240506B/donepezil hydrochloride, and UNC10100724A/ketoconazole were selected for the first round of dose‐response follow‐up because they stimulated phagocytosis in one of the cell lines tested with no or low associated cytotoxicity in the single concentration testing (Table 1).^10^ When we compare the data from Table 1 and Figure 1A for UNC10302865A, we see that the stimulation of microglial phagocytosis in HMC3 cells and inhibition of phagocytosis in BV2 cells predicted by the single‐concentration data (Table 1) was observed in dose‐response when tested up to 20 μM (top graphs) and was even more striking when the concentration range was expanded up to 40 μM (bottom graphs). Some toxicity was observed at the highest concentrations in BV2 cells. For UNC10240506B, the single‐concentration data predicted stimulation of phagocytosis in both cell lines (Table 1). Robust stimulation was observed in both cell lines when tested in dose‐response (Figure 1B) with toxicity limited only to the 40 μM dose in BV2 cells. For both UNC10302865A and UNC10240506B, there is clearly a dosing window between observed toxicity and a phenotypic response. Also, in both cases, the single‐point data was predictive of the dose‐response data.

**TABLE 1:** Summary of 10 μM single‐concentration data, normalized to control (DMSO) treated cells, for compounds selected for dose‐response follow‐up

		Cell number		Nuclear size		DNA intensity		Phagocytosis	
	Gene	HMC3	BV2	HMC3	BV2	HMC3	BV2	HMC3	BV2
UNC10302865A	ERBB3 (HER3)	132.1	102.4	105.8	110.1	90.8	101.0	152.7	68.8
UNC10240506B	ACHE	130.2	126.0	100.5	103.1	98.0	104.5	123.4	255.2
UNC10100724A	CYP3A43	125.5	129.7	96.3	103.8	100.1	104.7	144.4	280.0
UNC10244898A	ALK	127.2	117.2	110.3	102.7	89.2	110.1	37.8	200.3

**FIGURE 2:**
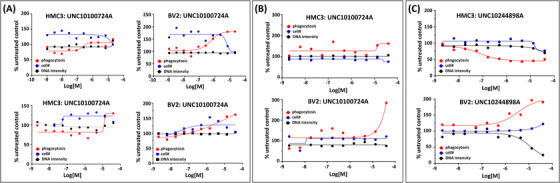
Phagocytosis assay 24‐ or 48‐hour dose‐response follow‐up for (A‐B) UNC10100724A and (C) UNC10244898A in HMC3 and BV2 cells

Like the compounds in Figure 1, UNC10100724A was dosed up to 20 μM (Figure 2A, top graphs) and up to 40 μM (Figure 2A, bottom graphs) for 24 hours. The same compound was also dosed up to 40 μM for 48 hours (Figure 2B). The single‐concentration data (Table 1) predicted stimulation of phagocytosis in both cell lines. This stimulation of phagocytosis was observed when cells were treated for either 24 or 48 hours with UNC10100724A in dose‐response and without notable toxicity. Finally, UNC10244898A was introduced in dose‐response up to 40 μM for 48 hours (Figure 2C). As shown in Table 1, UNC10244898A was predicted by single‐concentration data to inhibit phagocytosis in HMC3 cells and stimulate it in BV2 cells. Some toxicity was observed with UNC10244898A at the highest doses, but inhibition/stimulation of phagocytosis occurred at a much lower concentration. This provides a dosing window to elicit these changes in phagocytosis without associated toxicity. Mechanisms driving the observed differential phagocytic responses to UNC10244898A in HMC3 and BV2 cells are unknown and warrant further study. This comparison of single‐concentration and dose‐response data in our phagocytosis assay gives us confidence that the single‐concentration data can be used to reliably predict a phagocytic phenotype using our assay system.

**FIGURE 3:**
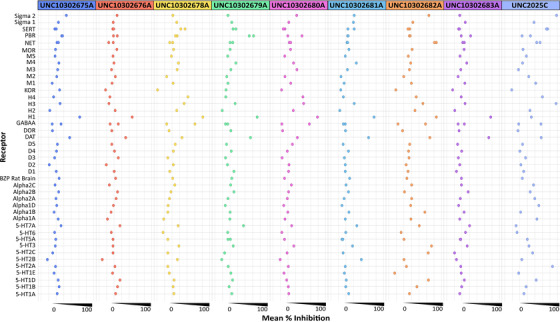
Visualization of G‐protein coupled receptor primary binding data for selected AD Informer Set compounds

Analysis of the GPCR panel screening results yielded many interesting findings (Figure 3). UNC10302682A/P505‐15 and UNC2025C possessed affinity for the most receptors in primary assays and were thus analyzed in the most secondary assays. Confirmed affinity (via secondary assays) was only observed for a portion of the receptors identified in the primary assays. UNC10302682A possessed modest affinity (calculated Ki values) for the Sigma2 (3.0 μM), Alpha1A (5.1 μM), H1 (1.2 μM), 5‐HT2C (7.2 μM), 5‐HT2A (7.2 μM), 5‐HT1D (3.3 μM), and 5‐HT3 (1.9 μM) receptors, and the norepinephrine and dopamine transporters (1.0 and 2.1 μM, respectively). In contrast, UNC2025C possessed affinity for the Sigma2 (0.2 μM), Alpha1D (5.0 μM), H4 (4.0 μM), and 5‐HT2A (0.4 μM) receptors, and the norepinephrine, serotonin, and dopamine transporters (1.4, 0.9, and 9.9 μM, respectively). While these compounds demonstrated affinity for several GPCRs, it is worth noting that their on‐target activity provides a large window at which to dose without engaging these peripheral receptors. The Ki of UNC2025C for MerTK, for example, is ≈200 pM while the Ki values observed in the secondary GPCR assays were in the micromolar range.^26^ Because UNC10302682A and UNC10302678A/entospletinib are both inhibitors of SYK enzymatic activity, the GPCR affinity observed for UNC10302682A does not seem tied to SYK inhibition but rather to off‐target protein binding interactions. Similarly, another published SYK inhibitor (BI1002494) also lacked this activity when profiled against many of the same GPCRs.^27^ Interestingly, UNC10302682A and UNC2025C are both protein kinase inhibitors. Their structures, however, are not very similar. With a few exceptions, compounds targeting EPHX2, CAPN2, and MDK proteins did not exhibit high affinity in secondary binding assays.

**FIGURE 4:**
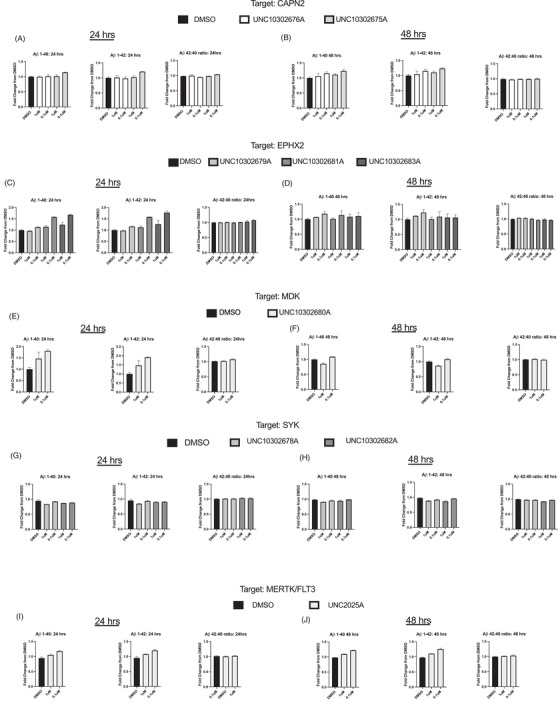
Amyloid beta (Aβ) peptide secretion assay results for (A‐B): UNC10302676A and UNC10302675A; (C‐D): UNC10302679A, UNC10302681A, UNC10302683A; (E‐F): UNC10302680A; (G‐H): UNC10302678A, UNC10302682A; (I‐J): UNC2025C. Aβ40 is in the left panel, Aβ42 in the middle panels, and the Aβ42:40 ratio in the bottom panel. Indicated are 24 hours (A, C, E, G, I) and 48 hours (B, D, F, H, J) treatment times

Our initial iPSC‐based neuronal studies provide an example of testing AD Informer Set compounds in primary cell assays. The cell line chosen for these studies, *APOE* ε3/ε4, harbors the most common risk for AD development and is representative of a patient genome that could develop sporadic AD. Aβ40 and Aβ42 are secreted in response to the three major cleavages within the transmembrane domain of amyloid precursor protein (APP).^28^ While Aβ40 is more prevalent, the relative ratios of Aβ42:40 can be calculated to determine whether there is a specific effect of a compound on γ‐secretase. For example, some familial AD (FAD) mutations in presenilin‐1 (PS1) cause a change in γ‐secretase cleavage such that Aβ42 peptides increase and Aβ40 peptides decrease.^29^ However, for this study, we did not observe a change in the Aβ42:40 ratio upon treatment (Figure 4A‐J). In some cases, we detected fluctuations in individual Aβ peptide levels, for example with UNC10302679A/GSK2256294A, UNC10302681A/TPPU, UNC10302683A/AR9281, and UNC10302680A/iMDK at 24 hours (Figure 4C and 4E); however, by 48 hours of treatment most of these had resolved (Figure 4D and 4F). In other cases we did not observe changes in individual Aβ peptides (Figure 4A, 4B, 4G‐J).

The Aβ secretion results (Figure 4) demonstrated that compounds targeting CAPN2, EPHX2, MDK, SYK, or MerTK/FLT3 proteins do not have a strong effect on Aβ40, Aβ42, or the resultant ratio of the two peptides. This finding was true at both concentrations and at both timepoints examined. In a previous report, UNC10302675A/MDL 28170 did not inhibit the secretion of Aβ40 and Aβ42 in N2a cells stably expressing wild‐type PS1 and myc‐tagged Swedish mutant APP (APPsw) when dosed at 30 μM for 24 hours. Levels of secreted Aβ40 and Aβ42 increased dramatically between 24 and 48 hours at this concentration. At concentrations < 5 μM, treatment for 48 hours did not result in a response in secreted Aβ40 or Aβ42, supporting our results.^28^ EPHX2 enzyme inhibitors have been reported to prevent the cytotoxicity induced by Aβ peptides, but their effect on modulating secreted Aβ levels was not examined.^20^ The same is true for MDK protein, which binds directly to Aβ40 to inhibit its cytotoxicity in vitro.^30^ SYK inhibitor BAY61‐3606 (2 mg/kg) stimulated transport of Aβ40 and Aβ42 in transgenic PS1/APPsw mice, resulting in a significant reduction in the detectable levels of both peptides.^31^

**FIGURE 5:**
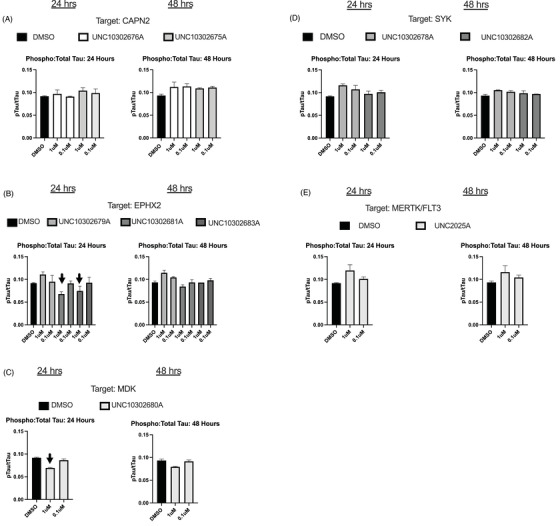
Phosphorylated tau (p‐tau):total tau (t‐tau) ratio results for (A): UNC10302676A and UNC10302675A; (B): UNC10302679A, UNC10302681A, UNC10302683A; (C): UNC10302680A; (D): UNC10302678A, UNC10302682A; (E): UNC2025C. UNC10302681A, UNC10302683A, and UNC10302680A lowered the p‐tau:t‐tau ratio at the 1 μM dose after 24 hours of treatment (panels B and C, indicated by arrows); 24‐ and 48‐hour timepoints are indicated

Excessive phosphorylation of tau is a hallmark for AD and thus agents that reduce tau phosphorylation are sought. A decrease in the ratio of p‐tau:t‐tau suggests a decrease in tau phosphorylation. In this study, when we calculated the p‐tau:t‐tau ratios, we observed that several compounds reduced the p‐tau:t‐tau ratio in the neurons (Figure 5B and C, indicated by arrows). In particular, a reduced ratio was observed for UNC10302681A/TPPU, UNC10302683A/AR9281, and UNC10302680A/iMDK when dosed at 1 μM for 24 hours (Figure 5C). This decrease was not detected at the lower concentration after 24 or at 48 hours. UNC10302681A and UNC10302683A target the EPHX2 enzyme, while UNC10302680A is an inhibitor of the MDK pathway. In support of our results, treatment of AD model mice with UNC10302681A (5 mg/kg/day) resulted in a reduction of tau hyperphosphorylation species (Ser396 and Ser404).^32^ Ebselen, an irreversible inhibitor of the EPHX2 enzyme, has also been shown to decrease tau phosphorylation in a triple transgenic AD mouse model.^33^, ^34^ It has been suggested that oxidative stress can promote tau hyperphosphorylation and resultant aggregation and that inhibition of the EPHX2 enzyme is anti‐inflammatory.^32^ Less has been published establishing a connection between MDK and tau phosphorylation. No effect on the p‐tau:t‐tau ratio was observed at either concentration or timepoint for compounds targeting CAPN2, SYK, or MerTK/FLT3 proteins (Figure 5A, 5D, 5E).

**FIGURE 6:**
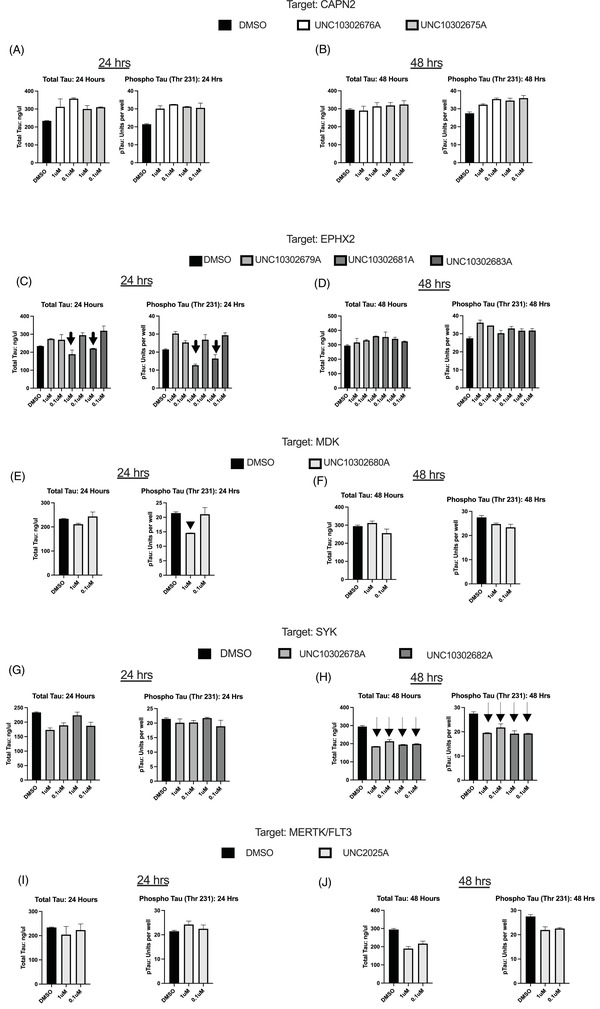
Results for individual levels of phosphorylated (p‐tau) and total tau (t‐tau) peptides for (A‐B): UNC10302676A and UNC10302675A; (C‐D): UNC10302679A, UNC10302681A, UNC10302683A; (E‐F): UNC10302680A; (G‐H): UNC10302678A, UNC10302682A; (I‐J): UNC2025C. UNC10302681A and UNC10302683A lowered both p‐tau and t‐tau with a larger effect on the p‐tau levels (panel C, indicated by arrows). UNC10302680A reduced the p‐tau levels with little effect on t‐tau (panel E, arrowhead) and UNC10302682A reduced p‐tau and t‐tau peptides to the same extent (panel H, dashed arrows). 24‐ and 48‐hour timepoints are indicated

We further examined the total levels of each peptide (Figure 6). Interestingly, at the higher 1 μM dose, EPHX2 enzyme inhibitors UNC10302681/TPPU and UNC10302683A/AR9281 decreased both p‐tau and t‐tau but the decrease in p‐tau was more pronounced, resulting in an observed shift in the ratio (Figure 6C, indicated by arrows). However this effect was not present at 48 hours of treatment (Figure 6D). UNC10302680A/iMDK also showed a larger reduction in p‐tau (Figure 6E, indicated by an arrowhead). While this was observed at 24 hours, the effect was not as significant at 48 hours (Figure 6F), which leads us to suggest that it is not due to toxicity of the compounds. With SYK enzyme inhibitors, we observed a decrease in the levels of p‐tau and t‐tau at 48 hours with both doses (Figure 6H, indicated by dashed arrows), but not at 24 hours (Figure 6G). This aligns with previous reports showing that SYK inhibition leads to decreased tau expression and a reduction in p‐tau.^35^ It was suggested that inhibition of SYK with BAY61‐3606 indirectly reduces tau phosphorylation via GSK3β and/or PI3K inhibition, but this result was only observed at higher concentrations than we tested (10 μM).^31^ In our treatments, both t‐tau and p‐tau were decreased to the same extent, therefore not changing the p‐tau:t‐tau ratio. While FLT3 has been implicated as a kinase that phosphorylates tau in vitro, there is no report of FLT3 inhibitors impacting tau phosphorylation and we noticed no effect at 24 hours and only a modest effect at 48 hours with MERTK/FLT3 inhibitors (Figure 6I and 6J).^36^ Although we did not observe changes in t‐tau or p‐tau with CAPN2 inhibitors (Figure 6A and 6B), similar to the reported mechanism for SYK, an indirect link has been proposed connecting CAPN2 protein activation and tau phosphorylation in AD via CDK5 activation.^37^

Our iPSC‐derived neuronal model provides a platform through which these compounds can be screened for effects on AD‐related peptides in a biologically relevant system. It is our goal that these preliminary results will provide baseline information for other investigators to perform more detailed analyses with these compounds. To further probe our findings, we will use the RNA harvested from this experiment and use quantitative real‐time polymerase chain reaction to examine specific target genes and their associated pathways.

The mouse PK experiments, summarized in Table 2, taught us more about specific compounds in the AD Informer Set. First, UNC10302681A/TPPU demonstrated excellent PK with a long half‐life (T_1/2_ = 13.8 hours) and low clearance (1.3 mL/min/kg). In contrast, UNC10302683A/AR9281 revealed poor PK with a short half‐life (T_1/2_ = 0.24 hours) and high clearance (93 mL/min/kg). Based on these findings, UNC10302681A was selected for follow‐up studies aimed at determining its brain permeability while UNC10302683A was excluded. The remaining four compounds sent for snapshot PK exhibited half‐life and clearance values in between these two. Because UNC10302678A/entospletinib and UNC10302680A/iMDK required formulation in NMP/PEG‐400 (10:90) to execute the snapshot PK studies and this formulation cannot be used in vivo, studies aimed at determining their brain penetration were not pursued. Despite its solubility issues, which were confirmed in kinetic solubility experiments,^10^ UNC10302678A is in Phase 2 clinical trials for hematological malignancies. UNC10302680A also demonstrated poor kinetic solubility but has been dosed in mice.^38^ UNC10302681A has been dosed systemically in many preclinical animal models of chronic inflammation, while UNC10302683A, despite its short half‐life, is in Phase 2 clinical trials for patients with hypertension and impaired glucose tolerance.^39^, ^40^, ^41^ UNC10302679A/GSK2256294A and UNC10302682A/P505‐15 were also advanced as far as Phase 2 clinical trials for insulin resistance and subarachnoid hemorrhage (UNC10302679A), and rheumatoid arthritis (UNC10302682A).

**TABLE 2:** Summary of published and experimentally determined IV mouse PK values

Compound ID	Dose (mg/kg)	T_1/2_ (h)	C_max_ (μM)	AUC_last_ (h*μM)	CL (mL/min/kg)	Vss (L/Kg)	Brain: plasma at 1 hour (10 mg/kg)
UNC10302676A*	2.0	6	–	35.6	7.8	1.0	0.015
UNC10302675A*	3.0	0.74	2345	1184	41.8	1.23	0.10
UNC2025C*	3.0	3.8	4.36	9.78	9.22	2.33	1.96
UNC10302679A	3.0	0.64	4011	1892	26.3	0.62	0.018
UNC10302681A	3.0	13.8	2144	7149	1.31	1.55	0.46
UNC10302683A	3.0	0.24	1832	537	93.1	0.47	ND
UNC10302680A	3.0	1.16	1569	1240	37.8	2.61	ND
UNC10302682A	3.0	1.64	595	831	49.7	6.26	0.08
UNC10302678A	3.0	0.90	926	1674	28.5	2.34	ND

Abbreviations: AUC_max_, maximal area under the curve (drug concentration as a function of time); Brain:Plasma, brain to plasma ratio; CL, drug clearance; C_max_, highest concentration of drug measured; ID, identifier; ND, not determined; PK, pharmacokinetic; T_1/2_, half‐life; Vss, steady state volume of distribution.

Notes: *Published PK values and experimentally determined Brain:Plasma ratios.

Studies aimed at experimentally determining the brain exposure at 1 hour for compounds with published and acceptable PK highlighted UNC2025C as the most promising. UNC2025C demonstrated a brain:plasma ratio of 1.96.^42^ All other compounds demonstrated brain:plasma ratios < 1 and ranged from 0.015 for UNC10302676A/ABT‐957 to 0.46 for UNC10302681A/TPPU. For the compounds targeting the EPHX2 enzyme (UNC10302679A/GSK2256294A, UNC10302681A/TPPU, and UNC10302683A/AR9281), no BBB data was found for UNC10302679A, but UNC10302681A and UNC10302683A were reported as brain penetrant.^20^, ^32^, ^39^, ^40^, ^43^ The brain:plasma ratio of UNC10302681A when dosed orally in mice at 3 mg/kg was reported as 0.18.^20^, ^44^ Baboon positron emission tomography studies were performed with blocking doses of UNC10302683A and levels in the brain versus plasma quantified as well as specific brain regions imaged.^43^ No reported BBB data were found for the compounds targeting SYK (UNC10302682A/P505‐15) or CAPN2 (UNC10302676A/ABT‐957 and UNC10302675A/MDL 28170) proteins.

With these experimental values in hand, we evaluated three methods used to predict BBB penetration: CNS MPO (MultiParameter Optimization),^45^ the StarDrop software program,^46^ and the ADMET Predictor software program.^47^ CNS MPO, developed and used by scientists at Pfizer, is calculated based upon six parameters that have been correlated to improve brain penetration: ClogP, ClogD, molecular weight, topological polar surface area, number of hydrogen bond donors, and most basic center (pKa). Compounds with a CNS MPO score ≥4 are more likely to be BBB permeable.^45^ StarDrop is software for small molecule design and optimization that uses compound physicochemical properties to predict the concentration of compound in the brain versus blood as an expectation of BBB penetration (logBB) based on a defined compound training set.^46^ ADMET Predictor is a machine learning software tool that also predicts based on a training set whether a compound will penetrate the BBB plus a calculation of the logarithm of the brain/blood partition coefficient (logBB).^47^ The literature reports a range of logBB cutoffs from –1.00 to 0.63 to classify compounds as BBB penetrant, with many agreeing on logBB ≥ 0.00 predicting BBB penetrance.^48^, ^49^, ^50^

As seen in Table 3, the predictive methods did not always agree, which reflects the different methods and training sets used. When using a logBB threshold of ≥ 0.00, only UNC10302681A/TPPU and UNC2025C were suggested to be brain penetrant by one of the three methods, and the rank order of brain:plasma ratio was not accurately predicted by any of them. Gratifyingly, UNC10302681A and UNC2025C showed the highest experimentally determined brain:plasma ratios. We suggest that consideration of calculated scores from multiple sources is the optimal practice moving forward, with the understanding that if two predict brain permeability then chances of experimental verification are increased.

**TABLE 3:** Experimental versus calculated BBB penetration values

Compound ID	Brain:Plasma at 1 hour (10 mg/kg)	MPO score	StarDrop: LogBB value	ADMET Predictor: LogBB value
UNC2025C	1.96	2.7	−0.1211	0.25
UNC10302676A	0.015	3.8	−1.006	−0.438
UNC10302675A	0.10	3.3	−0.2819	−0.408
UNC10302679A	0.018	2.6	−0.6425	−0.386
UNC10302681	0.46	5.3	−0.4012	−0.261
UNC10302682	0.08	2.5	−0.7396	−0.237

Abbreviations: BBB, blood–brain barrier; Brain:Plasma, brain to plasma ratio; ID, identifier; LogBB, logarithmic ratio between the concentration of a compound in brain and blood; MPO, MultiParameter Optimization score.

As proof‐of‐concept and to demonstrate its utility, we used AD Informer Set compounds to screen compounds that modulate putative targets implicated in AD. We have analyzed AD Informer Set compounds in a diverse biochemical panel of GPCR assays, cell‐based assays involving immortalized mouse and human cells as well as human iPSC‐derived neurons, and mice. Some unanticipated results were obtained, which have prompted follow‐up studies and generated new hypotheses about the protein targets. Based on our results, it is suggested that some compounds could be used to transiently phenocopy AD pathology, while others have illuminated a new therapeutic direction to pursue.

## CONFLICTS OF INTEREST

Jeffrey Aubé has received compensation from the American Chemical Society, Elsevier Beillstein, Pergament & Cepeda, and the National Institutes of Health as well as consulting fees from the University of Kansas and an honorarium from Case Western Reserve University. He has also served as NIH study section chair twice in the past 36 months and held an unpaid leadership role for the ACS Division of Organic Chemistry. Kevin J. Frankowski received support as a speaker from the Gordon Research Conference. Timothy I. Richardson is an advisor for Enveda Biosciences and consults with AlphaSights. Xiaodong Wang received an honorarium and travel support from University of Pittsburgh. Stephen V. Frye received consulting fees from Artios, Astex, GSK‐Crick, Cullgen, Design Therapeutics, Flare, Larkspur, Mitokinin, Pathios, ReViral, Meryx, and eFFector. He has also received honoraria from NIEHS, Scripps, St. Jude, Emory/Winship Cancer Center, Oregon Health Sciences University, University of New Mexico Comprehensive Cancer Center, University of Lexington, and University of Utah. Stephen Frye also serves on the UNC CTSA study section as a reviewer. Carrow Wells received funding from the Mark and Chordoma Foundations to support attending meetings and/or travel. Jessica E. Young received travel reimbursements from AAIC, Columbia University, and Duke University. Jeffrey Aubé, Ivie L. Conlon, Kevin J. Frankowski, Dmitri B. Kireev, Timothy I. Richardson, Xiaodong Wang, Carrow Wells, Timothy M. Willson, Stephen V. Frye, and Alison D. Axtman disclose that they have patents planned, issued, or pending within the past 36 months but that there is no overlap of these patents with the work described herein. All funding provided to the institution and individual authors has been disclosed. No other authors have conflicts of interest to disclose.

## Supporting information

Supporting Information

Supporting Information

Supporting Information

## References

[1] Jones LH , Bunnage ME . Applications of chemogenomic library screening in drug discovery. Nat Rev Drug Discov. 2017;16:285‐296. 28104905 10.1038/nrd.2016.244

[2] Drewry DH , Wells CI , Andrews DM , et al. Progress towards a public chemogenomic set for protein kinases and a call for contributions. PLoS One. 2017;12:e0181585. 28767711 10.1371/journal.pone.0181585 PMC5540273

[3] Elkins JM , Fedele V , Szklarz M , et al. Comprehensive characterization of the Published Kinase Inhibitor Set. Nat Biotechnol. 2016;34:95‐103. 26501955 10.1038/nbt.3374

[4] Wells CI , Al‐Ali H , Andrews DM ,et al. The Kinase Chemogenomic Set (KCGS): an open science resource for kinase vulnerability identification. Int J Mol Sci. 2021;22:566. 10.3390/ijms22020566 PMC7826789 33429995

[5] Arrowsmith CH , Audia JE , Austin C ,et al. The promise and peril of chemical probes. Nat Chem Biol. 2015;11:536‐541. 26196764 10.1038/nchembio.1867 PMC4706458

[6] Müller S , Ackloo S , Arrowsmith CH , et al. Donated chemical probes for open science. Elife. 2018;7:e34311. 29676732 10.7554/eLife.34311 PMC5910019

[7] Basu A , Bodycombe NE , Cheah JH , et al. An interactive resource to identify cancer genetic and lineage dependencies targeted by small molecules. Cell. 2013;154:1151‐1161. 23993102 10.1016/j.cell.2013.08.003 PMC3954635

[8] Clemons PA , Bittker JA , Wagner FF , et al. The use of informer sets in screening: perspectives on an efficient strategy to identify new probes. SLAS Discov. 2021:24725552211019410. 10.1177/24725552211019410 PMC8991386 34130532

[9] Drewry DH , Wells CI , Zuercher WJ , Willson TM . A perspective on extreme open science: companies sharing compounds without restriction. SLAS Discov. 2019;24:505‐514. 31034310 10.1177/2472555219838210 PMC6624833

[10] Potjewyd F , Annor‐Gyamfi JK , Aubé J , et al. AD Informer Set: chemical tools to facilitate Alzheimer's disease drug discovery. BioRxiv. 2021. 10.1101/2021.07.22.453404v1. PMC9019904 35475262

[11] Azam S , Haque ME , Jakaria M , Jo SH , Kim IS , Choi DK . G‐protein‐coupled receptors in CNS: a potential therapeutic target for intervention in neurodegenerative disorders and associated cognitive deficits. Cells. 2020;9:506. 10.3390/cells9020506 PMC7072884 32102186

[12] Huang Y , Thathiah A . Regulation of neuronal communication by G protein‐coupled receptors. FEBS Lett. 2015;589:1607‐1619. 25980603 10.1016/j.febslet.2015.05.007

[13] The Company of Biologists Ltd . A role for GPCRs in neurogenesis. Development. 2013;140:e2106.

[14] Penney J , Ralvenius WT , Tsai LH . Modeling Alzheimer's disease with iPSC‐derived brain cells. Mol Psychiatry. 2020;25:148‐167. 31391546 10.1038/s41380-019-0468-3 PMC6906186

[15] Dolmetsch R , Geschwind DH . The human brain in a dish: the promise of iPSC‐derived neurons. Cell. 2011;145:831‐834. 21663789 10.1016/j.cell.2011.05.034 PMC3691069

[16] Castello J , Ragnauth A , Friedman E , Rebholz H . CK2—An emerging target for neurological and psychiatric disorders. Pharmaceuticals. 2017;10:7. 10.3390/ph10010007 PMC5374411 28067771

[17] Tejera D , Heneka MT . Microglia in Alzheimer's disease: the good, the bad and the ugly. Curr Alzheimer Res. 2016;13:370‐380. 26567746 10.2174/1567205013666151116125012

[18] Hansen DV , Hanson JE , Sheng M . Microglia in Alzheimer's disease. J Cell Biol. 2018;217:459‐472. 29196460 10.1083/jcb.201709069 PMC5800817

[19] Rosenberger AFN , Morrema THJ , Gerritsen WH , et al. Increased occurrence of protein kinase CK2 in astrocytes in Alzheimer's disease pathology. J Neuroinflammation. 2016;13:4. 26732432 10.1186/s12974-015-0470-x PMC4702323

[20] Liang Z , Zhang B , Xu M , et al. 1‐Trifluoromethoxyphenyl‐3‐(1‐propionylpiperidin‐4‐yl) urea, a selective and potent dual inhibitor of soluble epoxide hydrolase and p38 kinase intervenes in Alzheimer's signaling in human nerve cells. ACS Chem Neurosci. 2019;10:4018‐4030. 31378059 10.1021/acschemneuro.9b00271 PMC7028313

[21] Young JE , Fong LK , Frankowski H , Petsko GA , Small SA , Goldstein LSB . Stabilizing the retromer complex in a human stem cell model of Alzheimer's disease reduces tau phosphorylation independently of amyloid precursor protein. Stem Cell Rep. 2018;10:1046‐1058. 10.1016/j.stemcr.2018.01.031 PMC5919412 29503090

[22] Frankowski H , Yeboah F , Berry BJ , et al. Knock‐down of HDAC2 in human induced pluripotent stem cell derived neurons improves neuronal mitochondrial dynamics, neuronal maturation and reduces amyloid beta peptides. Int J Mol Sci. 2021;22:2526. 33802405 10.3390/ijms22052526 PMC7959288

[23] DeRyckere D , Lee‐Sherick AB , Huey MG, et al. UNC2025, a MERTK small‐molecule inhibitor, is therapeutically effective alone and in combination with methotrexate in leukemia models. Clin Cancer Res. 2017;23:1481‐1492. 27649555 10.1158/1078-0432.CCR-16-1330 PMC5354980

[24] Lon HK , Mendonca N , Goss S ,, et al. Pharmacokinetics, safety, tolerability, and pharmacodynamics of alicapistat, a selective inhibitor of human calpains 1 and 2 for the treatment of Alzheimer disease: an overview of Phase 1 studies. Clin Pharmacol Drug Dev. 2019;8:290‐303. 30052328 10.1002/cpdd.598

[25] Markgraf CG , Velayo NL , Johnson MP , et al. Six‐hour window of opportunity for calpain inhibition in focal cerebral ischemia in rats. Stroke. 1998;29:152‐158. 9445345 10.1161/01.str.29.1.152

[26] Zhang W , DeRyckere D , Hunter D , et al. UNC2025, a potent and orally vioavailable MER/FLT3 dual inhibitor. J Med Chem. 2014;57:7031‐7041. 25068800 10.1021/jm500749d PMC4148167

[27] Lamb DJ , Wollin SL , Schnapp A , et al. BI 1002494, a novel potent and selective oral spleen tyrosine kinase inhibitor, displays differential potency in human basophils and B cells. J Pharmacol Exp Ther. 2016;357:554‐561. 27048659 10.1124/jpet.116.233155

[28] Dong Y , Tan J , Cui MZ , et al. Calpain inhibitor MDL28170 modulates Abeta formation by inhibiting the formation of intermediate Abeta46 and protecting Abeta from degradation. FASEB J. 2006;20:331‐333. 16354722 10.1096/fj.05-4524fje

[29] Tang N , Kepp KP . Aβ42/Aβ40 ratios of presenilin 1 mutations correlate with clinical onset of Alzheimer's disease. J Alzheimers Dis. 2018;66:939‐945. 30412504 10.3233/JAD-180829

[30] Yu GS , Hu J , Nakagawa H . Inhibition of beta‐amyloid cytotoxicity by midkine. Neurosci Lett. 1998;254:125‐128. 10214973 10.1016/s0304-3940(98)00685-5

[31] Paris D , Ait‐Ghezala G , Bachmeier C , et al. The spleen tyrosine kinase (Syk) regulates Alzheimer amyloid‐β production and tau hyperphosphorylation. J Biol Chem. 2014;289:33927‐33944. 25331948 10.1074/jbc.M114.608091 PMC4256331

[32] Griñán‐Ferré C , Codony S , Pujol E , et al. Pharmacological inhibition of soluble epoxide hydrolase as a new therapy for Alzheimer's disease. Neurotherapeutics. 2020;17:1825‐1835. 32488482 10.1007/s13311-020-00854-1 PMC7851240

[33] Domingues MF , Callai‐Silva N , Piovesan AR , Carlini CR . Soluble epoxide hydrolase and brain cholesterol metabolism. Front Mol Neurosci. 2020;12:325. 32063836 10.3389/fnmol.2019.00325 PMC7000630

[34] Xie Y , Tan Y , Zheng Y , Du X , Liu Q . Ebselen ameliorates β‐amyloid pathology, tau pathology, and cognitive impairment in triple‐transgenic Alzheimer's disease mice. J Biol Inorg Chem. 2017;22:851‐865. 28502066 10.1007/s00775-017-1463-2

[35] Schweig JE , Yao H , Coppola K , et al. Spleen tyrosine kinase (SYK) blocks autophagic tau degradation in vitro and in vivo. J Biol Chem. 2019;294:13378‐13395. 31324720 10.1074/jbc.RA119.008033 PMC6737214

[36] Cavallini A , Brewerton S , Bell A , et al. An unbiased approach to identifying tau kinases that phosphorylate tau at sites associated with Alzheimer disease. J Biol Chem. 2013;288:23331‐23347. 23798682 10.1074/jbc.M113.463984 PMC3743503

[37] Kurbatskaya K , Phillips EC , Croft CL , et al. Upregulation of calpain activity precedes tau phosphorylation and loss of synaptic proteins in Alzheimer's disease brain. Acta Neuropathol Commun. 2016;4:34. 27036949 10.1186/s40478-016-0299-2 PMC4818436

[38] Hao H , Maeda Y , Fukazawa T , et al. Inhibition of the growth factor MDK/midkine by a novel small molecule compound to treat non‐small cell lung cancer. PLoS One. 2013;8:e71093. 23976985 10.1371/journal.pone.0071093 PMC3745462

[39] Ulu A , Inceoglu B , Yang J , et al. Inhibition of soluble epoxide hydrolase as a novel approach to high dose diazepam induced hypotension. J Clin Toxicol. 2016;6:1000300. 28255523 10.4172/2161-0495.1000300 PMC5328659

[40] Minaz N , Razdan R , Hammock BD , Goswami SK . An inhibitor of soluble epoxide hydrolase ameliorates diabetes‐induced learning and memory impairment in rats. Prostaglandins Other Lipid Mediat. 2018;136:84‐89. 29751149 10.1016/j.prostaglandins.2018.05.004 PMC6402559

[41] Ulu A , Appt S , Morisseau C , et al. Pharmacokinetics and in vivo potency of soluble epoxide hydrolase inhibitors in cynomolgus monkeys. Br J Pharmacol. 2012;165:1401‐1412. 21880036 10.1111/j.1476-5381.2011.01641.x PMC3372725

[42] Wu J , Frady LN , Bash RE , et al. MerTK as a therapeutic target in glioblastoma. Neuro‐Oncol. 2017;20:92‐102. 10.1093/neuonc/nox111 PMC5761530 28605477

[43] Du Y , Minn I , Foss C , et al. PET imaging of soluble epoxide hydrolase in non‐human primate brain with [18F]FNDP. EJNMMI Res. 2020;10:67. 32572592 10.1186/s13550-020-00657-7 PMC7310027

[44] Ren Q , Ma M , Ishima T , et al. Gene deficiency and pharmacological inhibition of soluble epoxide hydrolase confers resilience to repeated social defeat stress. Proc Natl Acad Sci U S A. 2016;113:E1944‐E1952. 26976569 10.1073/pnas.1601532113 PMC4822604

[45] Wager TT , Hou X , Verhoest PR , Villalobos A . Moving beyond rules: the development of a Central Nervous System Multiparameter Optimization (CNS MPO) approach to enable alignment of druglike properties. ACS Chem Neurosci. 2010;1:435‐449. 22778837 10.1021/cn100008c PMC3368654

[46] https://www.optibrium.com/stardrop/

[47] https://www.simulations‐plus.com/software/admetpredictor/

[48] Carpenter TS , Kirshner DA , Lau EY , Wong SE , Nilmeier JP , Lightstone FC . A method to predict blood‐brain barrier permeability of drug‐like compounds using molecular dynamics simulations. Biophys J. 2014;107:630‐641. 25099802 10.1016/j.bpj.2014.06.024 PMC4129472

[49] Luco JM . Prediction of the brain‐blood distribution of a large set of drugs from structurally derived descriptors using partial least‐squares (PLS) modeling. J Chem Inf Comput Sci. 1999;39:396‐404. 10192950 10.1021/ci980411n

[50] Adenot M , Lahana R . Blood‐brain barrier permeation models: discriminating between potential CNS and non‐CNS drugs including P‐glycoprotein substrates. J Chem Inf Comput Sci. 2004;44:239‐248. 14741033 10.1021/ci034205d

