# Supplementary material for: Use of AD Informer Set compounds to explore validity of novel targets in Alzheimer's disease pathology
Source: Alzheimers Dement (N Y). 2022 Apr 12;8(1):e12253. doi: 10.1002/trc2.12253 (PMC9005681; doi:10.1002/trc2.12253)
Supplement: Supplementary file 1 — Supporting Information [file TRC2-8-e12253-s003.pdf]

Experimental details

Microglial viability and phagocytosis studies: The pHrodo-myelin phagocytosis/cell viability assay, modified based on a published method [1], was run with BV2 (mouse microglia) and HMC3 (human microglia) immortalized cell lines in the Chu lab at IUSM. The 384-well plate high content analysis assay with BV2 and HMC3 microglial cell lines is used to quantify phagocytosis and cell viability simultaneously. High content imaging is performed with the ArrayScan automatic imaging system and imaging analysis done with the system's software. Cells are treated with compounds for total 48 hours including final 20 hours seeded with pHrodo-myelin (labeled phagocytosis ligand, purified from mouse brain), then cells are stained with Hoechst-33342 one hour before the imaging. Three measurements are extracted from the assay: 1) phagocytosis signal by mean total phagocytosis spot intensity per cell, 2) total cell counts per well as the main measurement of cell viability, and 3) mean average nuclear intensity per cell as profiling of cell health since apoptotic cells showing nuclear intensity increase (early apoptosis) and decrease (later apoptosis), respectively.

Based on the single-concentration data, several compounds were selected for 10-point dose-response follow-up in duplicate or triplicate. These compounds were initially dosed up to 20 µM for 24h. It was clear that trends were emerging at the highest doses, so then the dose range was expanded up to 40 µM for 24h for a second round of testing. Finally, a subset of compounds was dosed up to 40 µM for 48h, allowing time for some targets to respond to treatment and changes in gene expression to manifest.

GPCR panel: Screening against the receptors within the PDSP panel offers an assessment of off-target pharmacological activity at cloned human or rodent CNS receptors, channels, and transporters that could confound CNS-related phenotypes. A total of 46–47 primary binding assays were run in quadruplicate on these compounds at 10 µM, with follow-up secondary binding assays in 11-point dose-response in triplicate for select compounds versus select receptors [2]. Secondary binding assays were executed for all receptors where test compound displaced an average of >50% of radio-labeled standard ligand. As an exception, no secondary assays were carried out for H3, PBR, or GABAA.

Human iPSC-derived cellular assays: Neurons derived from APOE e3/e4 cells [3, 4] were treated with either 0.1 or 1 µM of compound for 24h or 48h, and media, cell lysates, and RNA were harvested. Basal secretion of Aβ peptides (Aβ40 and Aβ42) by iPSC-derived neurons into culture media was assessed at two time points (24h and 48h) using a multiplexed ELISA assay. We calculated the fold-change from the vehicle control (DMSO) measured for Aβ40 and Aβ42. To look at the relationship of these two peptides to each other, we calculated the Aβ42:40 ratio for each compound.

Measurements of phospho-Tau (pTau, Thr231) and total Tau peptides were measured from neuronal lysates, also using a multiplexed ELISA assay. Using this assay, we measured the phospho:total tau ratio for each condition compared to vehicle (DMSO) treatment as well as the individual changes in phospho and total Tau peptides. Finally, all neuronal samples were examined morphologically for evidence of cytotoxicity.

PK data: For snapshot PK studies, pooled plasma was analyzed at 0.5h, 1h, 2h, and 4h following a single IV dose (3 mg/kg) in CD1 mice (2 mice per cohort) for each compound (Table 2). Compounds with acceptable PK were dosed IV at 10 mg/kg and plasma plus brain concentrations measured at 1h in CD1 mice (3 mice per cohort, Table 2).

[1] Andreone BJ, Przybyla L, Llapashtica C, Rana A, Davis SS, van Lengerich B, et al. Alzheimer's-associated PLCγ2 is a signaling node required for both TREM2 function and the inflammatory response in human microglia. Nat Neurosci. 2020;23:927-38.
[2] Besnard J, Ruda GF, Setola V, Abecassis K, Rodriguiz RM, Huang XP, et al. Automated design of ligands to polypharmacological profiles. Nature. 2012;492:215-20.
[3] Young JE, Fong LK, Frankowski H, Petsko GA, Small SA, Goldstein LSB. Stabilizing the retromer complex in a human stem cell model of Alzheimer's disease reduces tau phosphorylation independently of amyloid precursor protein. Stem Cell Rep. 2018;10:1046-1058.
[4] Frankowski H, Yeboah F, Berry BJ, Kinoshita C, Lee M, Evitts K, et al. Knock-down of HDAC2 in human induced pluripotent stem cell derived neurons improves neuronal mitochondrial dynamics, neuronal maturation and reduces amyloid beta peptides. Int J Mol Sci. 2021;22:2526.
